# Opposite trends in hospitalization and mortality after implementation of a chronic care model-based regional program for the management of patients with heart failure in primary care

**DOI:** 10.1186/s12913-018-3164-0

**Published:** 2018-05-30

**Authors:** Piercarlo Ballo, Francesco Profili, Laura Policardo, Lorenzo Roti, Paolo Francesconi, Alfredo Zuppiroli

**Affiliations:** 1 Cardiology Unit, S. Maria Annunziata Hospital, via dell’Antella 58, Florence, Italy; 2 Regional Health Agency of Tuscany, Florence, Italy; 3 Tuscany Region, Florence, Italy

**Keywords:** Heart failure, Health services, Mortality, Hospitalization, Chronic disease

## Abstract

**Background:**

The chronic care model (CCM) is an established framework for the management of patients with chronic illness at the individual and population level. Its application has been previously shown to improve clinical outcome in several conditions, but the prognostic impact of CCM-based programs for the management of patients with chronic heart failure (HF) in primary care is still to be elucidated.

**Methods:**

We assessed the prognostic impact of a primary-care, CCM-based project applied in Tuscany, Italy, in 1761 patients with chronic HF enrolled in a retrospective matched cohort study. The project was based on predefined working teams including general practitioners and nurses, proactively scheduled regular follow-up visitations for each patient, counseling for therapy adherence and lifestyle modifications, appropriate diagnostic and therapeutic pathways according to international guidelines, and a key supporting role of the nurses, who were responsible for the practical coordination of the follow-up. A matched group of 3522 HF subjects assisted by general practitioners not involved in the project was considered as control group. The endpoints of this study were HF hospitalization and all-cause mortality.

**Results:**

Over a 4-year follow-up period, HF hospitalization rate was higher in the CCM group than the controls (12.1 vs 10.3 events/100 patient-years; incidence rate ratio 1.15[1.05-1.27], *p* = 0.0030). Mortality was lower in the CCM group than the controls (10.8 vs 12.6 events/100 patient-years; incidence rate ratio 0.82[0.75-0.91], *p* < 0.0001). In multivariable analysis, the CCM status was associated with a 34% higher risk of HF hospitalization and 18% lower risk of death (*p* < 0.0001 for both). The effect on HF hospitalization was mostly driven by a 50% higher rate of planned HF hospitalization.

**Conclusions:**

Implementation of a CCM-based program for the management of HF patients in primary care led to reduced mortality and increased HF hospitalization. These findings support the hypothesis that the beneficial effects of CCM on survival might be extended to patients with chronic HF followed in primary care, but also support the need for further strategies aimed at improving the management of these patients in terms of hospitalizations.

## Background

Despite the progressive reduction in cardiovascular death rates over the last decades, chronic heart failure (HF) remains a major and growing health system challenge worldwide, and a leading cause of mortality, recurrent hospitalization, and disability [1, 2]. Hospital discharges related to HF have progressively increased over the last decades in both US and Europe, and now exceed 1 million per year in the US [3–5]. To date, the incidence of HF in adults is 5–10 per 1000 persons per year in developed Countries, resulting in an overall prevalence of 2-3% [6, 7]. The impact of HF is even more evident in the elderly, exceeding a 10% prevalence among persons ≥70 years of age [8]. The prognosis of patients with HF also remains poor, with approximately 50% of patients expected to die within 5 years and with no significant trends towards improvement over the last two decades [9–12]. Moreover, despite the progressive advances in the pharmacological therapy of HF, gaps between guidelines and clinical practice in HF patients are still evident [13]. All these factors contribute to impose a huge and progressively growing economic burden on healthcare systems [14].

These considerations raise the question of whether the development and the implementation of specific management programs could be effective in improving the clinical outcome of patients with HF. The Chronic Care Model (CCM) is a well-known model aimed at transforming the health care system from simply reactive – i.e., responding in case of sickness - to a proactive one, thus focusing on the maintenance of patients’ health by planned regular interventions at the community, organization, practice, and patient levels [15, 16]. Although this model has been widely applied worldwide for the management of patients with chronic diseases, few analyses investigated its effectiveness in improving outcomes in HF patients, with considerable differences in the effects on hospitalization and quality of life across the studies [17–20]. A recent metanalysis confirmed that the CCM approach can probably be clinically useful for the management of HF patients, but with substantial heterogeneity in effectiveness [21]. Moreover, most of these studies were carried out in the US or in northern European Countries, whose health care systems are different from the Italian one [22, 23]. Lastly, the majority of evidences were obtained in hospital settings, so that the clinical utility of CCM-based programs for the management of HF patients in primary care is still to be elucidated. In this view, we sought to explore the effect of CCM on the outcome of HF patients within the Italian system, which is based on healthcare services provided by a public system administered on a regional basis and hinges on a central role of the general practitioner (GP). We hypothesized that the application of a CCM project in this setting could have lead a positive impact on clinical outcome. The rationale of this hypothesis was the assumption that a proactive approach, aimed at optimizing patient-related and particularly system-related factors, would have favoured a better adherence to guideline-recommended treatments. By investigating the potential role of CCM in a chronic disease with large prevalence and high economic burden, we also expected this study to contribute to the health service research field.

Therefore, the objective of this study was to investigate the clinical utility of a CCM-based healthcare project for the management of patients with HF.

## Methods

### Setting and intervention

This study was designed to explore the prognostic impact of a regional healthcare project applied since 2010 in Tuscany, Italy, aimed at optimizing the clinical management of patients with HF in primary care and based on the implementation of the CCM. The “Project for proactive health care implementation at community level” was launched in 2008 by the Tuscan Regional Health Ministry as a major target of the 2008-2010 Health Planning, and was based on the implementation of the CCM in several diseases, including HF. The project involved the whole population living in Tuscany, Italy, and was applied to patients with chronic HF since 2010. The present analysis is a retrospective matched cohort study on this population. The project was designed by taking into account the characteristics of the local healthcare system. Italy has a tax-based universal health system organized on several levels. The national level provides funding and dictates the fundamental services that must be provided to every inhabitant. The regional level receives the national funding and organizes the health system through a network of local health authorities. Every inhabitant is entitled to choose a GP, who has a gatekeeper function and may have in charge a maximum of 1500 adult subjects. GPs can work either as single physicians or functionally associated with other colleagues. Copayment of some health services may be requested, according to national or regional regulations. Local health authorities are further subdivided in health districts that are homogeneous with respect to several characteristics (e.g., rural vs urban vs mountain areas), and primary care is organized at the health district level.

For this project, GPs were organized in teams comprising 5 to 15 physicians and at least a nurse per 10,000 patients. The project was specifically designed to implement the main principles of the CCM for the management of chronic HF patients in primary care. Accordingly, regular follow-up visits were proactively scheduled for each patient and recalls were set up for patients who were not showing up. Particular care was given to provide adequate and systematic counselling for therapy adherence and lifestyle modifications – including regular physical activity, weight loss when appropriate, smoke cessation, and adequate dietary intake – and to establish an effective patient-provider relationship based on collaborative care and self-management education [24, 25]. The GPs adhered to the project on a voluntary basis. All the GPs who adhered were members of some form of association and adherence was always a groups’ decision. A pay for performance scheme was set up, based on the following indicators: percentage of patients who were enrolled, who were treated with ACE inhibitors/Angiotensin receptor blockers and beta-blockers, who had creatinine and electrolyte blood tests, who attended individual or group counselling, and who had their weight measured. The pay for performance scheme was the same throughout the study period. The dedicated nurse had a key role in the project, as she was responsible for updating the chronic disease registry, contacting patients for routine services, scheduling specialist visits, managing patient counselling, providing additional self-management support and health behaviour counselling, and systematically recording weight and blood pressure.

In each local health authority that participated to the project, diagnostic and therapeutic pathways were developed according to international guidelines compatible with the local available resources. GPs adhering to the project enrolled patients over a predefined six-month period, from January to June 2010.

### Study sample

**Fig. 1:**
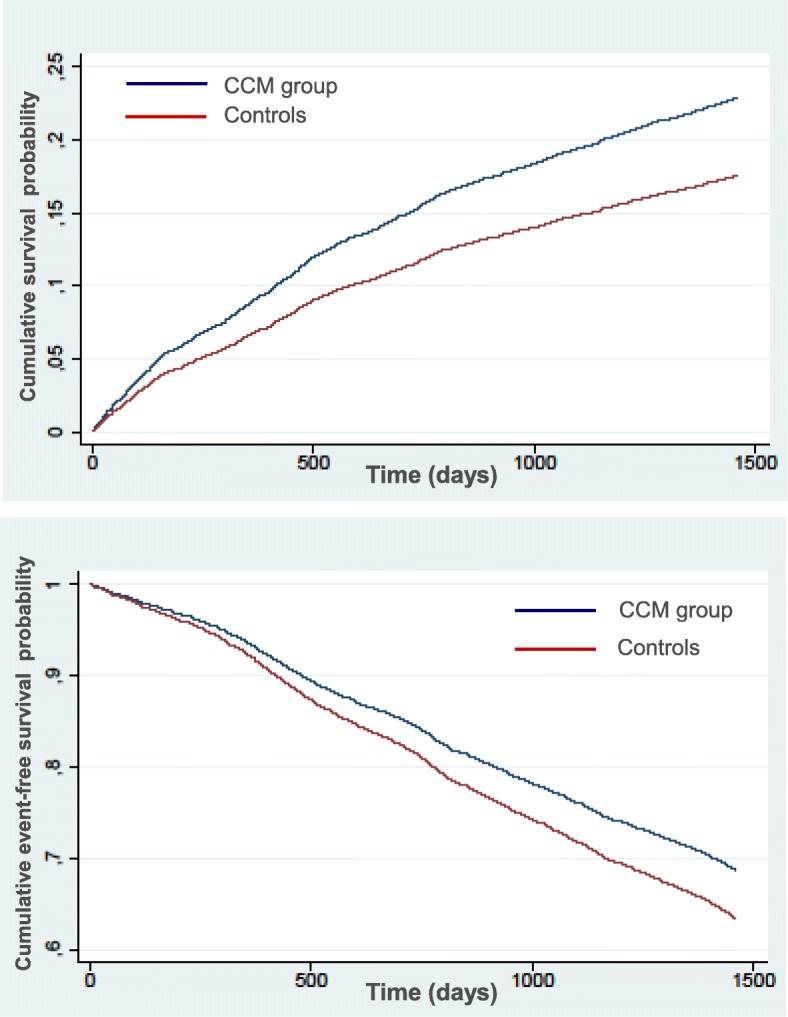
*Top panel*: Kaplan-Meier curves showing cumulative survival probability in the chronic care model (CCM, blue curve) group and in the control group (red curve). *Bottom panel*: Kaplan-Meier curves showing cumulative event-free survival probability in the chronic care model (CCM) group and in the control group

For the purpose of this study, the exposed cohort (CCM group) was selected among all patients enrolled in the CCM project by their GP because of chronic HF (Fig. 1, group A). Among them, only patients classified as having a definite diagnosis of chronic HF by administrative data (Fig. 1, group B) were included in the CCM group (Fig. 1, group C). The following administrative data were considered: one or more hospital discharges with primary ICD9 code indicating HF (428, 3981, 40201, 40211, 40291, 40401, 40403, 40411, 40413, 40491, 40493), exemption to payment because of chronic HF [26]. In this group, complete data were available for 1761 (94.3%) patients, which formed the final CCM study cohort. The unexposed cohort (control group) was selected among all patients with a diagnosis of HF by the same administrative data and who were assisted by GPs not adhering to the CCM project (Fig. 1, group D). To minimize the risk of selection bias, exposed and unexposed subjects were exactly matched with a 1:2 ratio for age class, gender, Charlson comorbidity index (a validated and widely used prognostic score related to the number and severity of comorbidities), geographic area of living (defined as the local health authority), treatment with ACE-inhibitors and/or ARBs, beta-blockers, and diuretics, and history of hospitalization for HF during the previous 5 years (between 2005 and 2009). We used a frequentist matching method, randomly selecting two unexposed subjects in each stratum given by the matching variable combinations. The final CCM and control group included 1761 and 3522 patients, respectively. All subjects were followed from the baseline (discharge) until death, readmission or end of follow-up period (4 years), whichever came first. No ethical approval was needed for this type of study. For patients in both groups, all data were extracted from an administrative archive (Health Informative Database of Tuscany Region, Italy), using an anonymous code to link subjects between different databases (hospitalizations, drugs, mortality).

### Endpoints

Two different endpoints were considered in this study: 1) hospitalization for HF; 2) all-cause mortality. The follow-up encompassed a 4-year period, from January 1, 2010 to December 31, 2013. Hospitalizations for HF were identified by considering all discharges for the Aggregate Clinical Code 108, which is obtained by grouping the following ICD-9 codes: 39891, 4280, 4281, 42820-42823, 42830-42833, 42840-42843, 429.

### Statistical analysis

Data for categorical variables were shown as numbers (percentages). Incidence rates and incidence rate ratios (IRR, exposed vs unexposed) were calculated for each clinical endpoint. Event-free survival analysis was performed by multivariable Cox regression, to assess the impact of CCM project on the risk of HF hospitalization and mortality. Analyses were carried out adjusting for death competing risk. For the endpoints of HF hospitalization and all-cause mortality, only the first event was considered in survival analyses. The age classes used in survival analyses were identified using predefined cut-offs (< 75, 75 to 85, and > 85 years). A *p* < 0.05 was considered significant. All analyses were performed using STATA, ver. 12 (STATA Corp., College Station, TX, USA).

## Results

### Main characteristics

Main characteristics of exposed and unexposed subjects are shown in Table 1. As a result of the matching procedure, the two groups showed equal percent distributions of age class, gender, Charlson index, use of main cardiovascular pharmacological classes, local health authority of living, and number of hospitalizations for HF during the 2005-2009 period. The median follow-up time was 1148 [1124-1174] days in the CCM group and 1045 [1024-1066] days in the controls. During the period of study, an increase in the proportions of subjects who were treated with beta-blockers and with ACE inhibitors/angiotensin receptor blockers, and who had creatinine and electrolyte blood tests checked was observed in both groups (Table 2). After adjusting for pre-intervention ones, the final values were significantly higher in the CCM group for beta-blocker therapy (IRR 1.07 [1.03-1.12], *p* < 0.0001) and for creatinine and electrolyte blood tests (IRR 1.20 [1.16-1.25], *p* < 0.0001).

**Table 1:** Main characteristics

	CCM group (*n* = 1761)	Control group (*n* = 3522)	Prevalence^a^
Female gender	763	1526	43.3%
Age class			
< 75	559	1118	31.7%
75-85	766	1532	43.5%
> 85	436	872	24.8%
Charlson comorbidity index			
0	592	1184	33.6%
1	366	732	20.8%
2	803	1606	45.6%
Treatment at enrolment^b^			
ACE-inhibitors or ARBs	1526	3052	86.7%
Beta-blockers	1036	2072	58.8%
Diuretics	1484	2968	84.3%
HF hospitalization between 2005 and 2009			
0	181	362	10.3%
1	322	644	18.3%
≥ 2	1258	2516	71.4%
Local Health Unit			
103	2	4	0.1%
104	121	242	6.9%
105	1	2	0.1%
106	124	248	7.0%
107	179	358	10.2%
108	268	536	15.2%
109	267	534	15.2%
110	687	1374	39.0%
111	74	148	4.2%
112	38	76	2.2%

Main characteristics of the chronic care model group. The *P* value was 1 for all comparisons, as a result of the matching procedure. *ARBs* angiotensin receptor blockers, *ACE* angiotensin converting enzyme, *HF* heart failure

^a^Equal prevalences in both groups as a result of the exact matching

^b^The majority of patients were treated with multiple medications at enrolment

**Table 2:** Process and therapeutic indicators

	CCM group (n = 1761)	Control group (n = 3522)
Creatinine and electrolyte tests		
Rate 2005-2009	55.8%	52.4%
Rate 2010-2013	80.7%	65.3%
Beta-blocker therapy		
Rate 2005-2009	45.0%	43.0%
Rate 2010-2013	65.1%	59.5%
ACE/ARBs therapy		
Rate 2005-2009	78.2%	76.7%
Rate 2010-2013	81.0%	80.2%

Rates of diagnostic and therapeutic indicators in the periods before and after chronic care model (CCM) project implementation among exposed and unexposed subjects. *ARBs* angiotensin receptor blockers, *ACE* angiotensin converting enzyme

### HF hospitalization

During the follow-up, there were 713 hospitalizations for HF in 432 patients within the.

CCM group (12.1 events per 100 patient-years) and 1135 hospitalizations in 657 patients within the control group (10.3 events per 100 patient-years), indicating a higher incidence in the CCM group than in the controls (IRR 1.15 [1.05-1.27], *p* = 0.0030). Mean length of stay was 8.8 days in the CCM group and 8.1 days in the controls, corresponding to 1.07 and 0.84 days per patient-year, respectively (IRR 1.25 [1.21-1.29], *p* < 0.0001). Multivariable analysis (Table 3) showed that CCM status was independently associated with 35% higher probability of HF hospitalization (HR 1.35, 95% CI 1.19-1.52, p < 0.0001). The curves showing the adjusted cumulative survival probability in the two groups are shown in Fig. 1, top panel.

**Table 3:** Event-free survival analysis

	Hazard ratio	95% CI	P value
CCM status	1.35	1.19-1.52	< 0.0001
Age class^a^			
75-85	1.40	1.21-1.63	< 0.0001
> 85	1.59	1.33-1.89	< 0.0001
Female gender	0.96	0.85-1.09	0.51
Charlson index	1.16	1.07-1.26	< 0.0001
Treatment at enrolment			
ACE-inhibitors or ARBs	1.09	0.90-1.31	0.38
Beta-blockers	1.24	1.09-1.41	0.0010
Diuretics	2.38	1.87-3.03	< 0.0001
Geographic area	1.00	0.97-1.03	0.99
Previous HF hospitalization	1.11	0.99-1.25	0.069

Predictors of hospitalization in the overall study population, as identified by multivariable Cox regression analysis. *ACE* angiotensin converting enzyme, *ARBs* angiotensin receptor blockers, *CCM* chronic care model, *CI* confidence interval

^a^Hazard ratios calculated vs age < 75 years as a reference

After a first hospitalization for HF, no difference was observed between the two groups in the risk of further HF hospitalizations (IRR 1.001 [0.89-1.13], *p* = 0.98). The rate of 30-day HF readmissions after a first HF hospitalization also showed no significant difference between the CCM group and the controls (4.9% vs 5.9%, *p* = 0.14).

When planned and urgent HF hospitalizations were considered separately, CCM status was associated with a significantly higher rate of planned hospitalizations (HR 1.50 [1.15-1.94], *p* < 0.0001). This effect was more evident than that observed on the rate of urgent hospitalizations (hospitalizations for HF: HR 1.29 [1.13-1.46], p < 0.0001).

### Mortality

There were 632 deaths in the CCM group (10.8 events per 100 patient-years) and 1393 deaths in the control group (12.6 events per 100 patient-years; IRR 0.82 [0.75-0.91], p < 0.0001). Univariable Cox regression in the overall population showed that CCM status was associated with 15% lower risk of death (HR 0.85, 95% CI 0.78-0.94, *p* = 0.001). Multivariable analysis confirmed that CCM status was independently associated with a 18% risk reduction in mortality (Table 4). Adjusted cumulative survival probabilities in the two groups are shown in Fig. 1, bottom panel. Interestingly, even after a hospitalization for HF, patients in the CCM group still showed a 16% lower risk of death than the controls (HR 0.84 [0.71-0.99], *p* < 0.05).

**Table 4:** Survival analysis

	Hazard ratio	95% CI	P value
CCM status	0.82	0.75-0.91	< 0.0001
Age class^a^			
75-85	1.89	1.67-2.15	< 0.0001
> 85	3.59	3.14-4.09	< 0.0001
Female gender	0.87	0.79-0.95	0.0020
Charlson index	1.39	1.31-1.48	< 0.0001
Treatment at enrolment			
ACE-inhibitors or ARBs	0.77	0.68-0.86	< 0.0001
Beta-blockers	0.81	0.74-0.89	< 0.0001
Diuretics	1.98	1.67-2.34	< 0.0001
Geographic area	1.01	0.98-1.03	0.62
Previous HF hospitalization	1.12	1.02-1.22	0.015

Predictors of death in the overall study population, as identified by multivariable Cox regression analysis. *ACE* angiotensin converting enzyme, *ARBs* angiotensin receptor blockers, *CCM* chronic care model, *CI* confidence interval

^a^Hazard ratios calculated vs age < 75 as a reference

## Discussion

HF is the most common cause of hospitalization in Western countries, particularly in patients over the age of 65, and represents a major challenge to the health care systems. In outpatients with chronic HF, a hospitalization is one of the strongest prognostic predictors for increased mortality, and unplanned readmissions arouse a high financial burden [27]. An adequate knowledge of the precipitants of rehospitalisation in these patients is therefore of major importance [28]. Besides classical clinical factors such as myocardial ischemia, atrial fibrillation, uncontrolled hypertension, and exacerbations of chronic obstructive pulmonary disease or infections, non-clinical determinants of hospitalization (e.g., inadequate access to follow-up care or medications and poor transitions of care) are progressively growing in importance [29]. In this regard, the implementation of strategies aimed at improving the quality of health care delivery for patients with chronic HF may be of clinical interest. Although the true prognostic impact of the CCM still lacks consistent evidence of benefit across all medical conditions [15, 30], a potential positive effect on clinical outcome has been reported in various chronic diseases [31–35]. This study explored the effect of a CCM-based, regional program for patients with chronic HF applied in primary care over a 4-year follow-up. Our findings show that patients enrolled in the program showed a lower risk of death but a higher risk of hospitalization for HF than a matched control population.

Previous studies, mostly performed in hospital settings, reported that the application of the CCM for the management of patients with chronic HF could lead to potential beneficial effects on outcome, although with some heterogeneity in effectiveness [17–21]. The improved survival observed in this study adds to these evidences by suggesting that these potential benefits might be extended to a chronic HF population followed in primary care. This finding could suggest a higher quality of care for the patients in the CCM group, and potentially better cooperation between cardiologists at the hospital and the GP. However, the finding of an opposite trend for HF hospitalization and mortality may be somewhat surprising. Interestingly, a similar discrepancy was also previously reported in the Veterans Affairs Health Care System, where mortality and HF hospitalization rates showed a definite trend in opposite directions [36]. Several explanations could be proposed for the lack of decline in HF hospitalization in our population. Since our CCM program involved primary care physicians - therefore being somewhat different from disease-centric systems of care/interventions as we know them from the hospital/specialist point of view - we can hypothesize that they improved their awareness of HF patients and tended to assess their clinical status following clinical pathways and using more facilities, including hospitalization. This hypothesis is supported by the evidence that the CCM status was associated with a 50% increase in the rate of planned HF hospitalizations, whereas the effect on the rate of urgent hospitalizations was considerably smaller. Also, while it cannot be excluded that the adjustment for death competing risk in our analysis was not able to completely remove the increased probability of hospitalization resulting from the increased number of survivors, the possibility that not every hospitalization must be considered as a poor outcome - especially from the patient’s point of view - should be considered. In a study of intensive primary care follow-up following a discharge for chronic diseases including HF, admissions actually increased though patients rated their health better [37]. With this in mind, it is also interesting to observe that no differences in the risk of further HF hospitalizations or the rate of 30-day HF readmissions were found between groups in our study.

Regardless of the factors underlying this divergent trend, the finding of an increase in hospitalization rates among patients enrolled in CCM project carried out in a primary care setting – where the access to specialist consultations is not so direct as in hospitals or other facilities – might support the potential clinical utility of multidisciplinary approaches to be integrated within primary care CCM models. The role of multimorbidity in affecting the risk of hospitalization in patients with chronic diseases is established, particularly in the elderly [38, 39]. Though our CCM project was not designed to directly involve specialists in patients’ management, it could be hypothesized that such a multidisciplinary strategy, by means of providing a larger number of diagnostic and therapeutic pathways to the referring general physician, might potentially reduce the need for hospitalization. These concepts could also be applied not only to the doctors involved in the management of HF patients, but also to the nurses, who represent a key component in any CCM-based health care system [40].

Our results might also have implications for the use of both hospitalization and rehospitalization as measures of quality of care, suggesting that they should be used with caution. The limitations of these outcome measures are established, including the need of a time window that must be appropriate for the type of disease, the effect of case-mix factors, the competing risk of mortality, and the fact that admissions must be avoidable and unplanned [41]. Also, the possibility of a residual confounding role of measured and unmeasured individual factors that affect the likelihood of hospitalization – not necessarily related to the quality of clinical care, such as social support, geographic location, and socioeconomics – should be taken into account [42, 43]. Accordingly, while most efforts at discharge commonly focus on managing congestion and close hemodynamic monitoring to reduce early readmissions, broader strategies to treat HF-related comorbidities and patient-centered management may probably be useful in the perspective of hospitalizations over a longer time [44, 45].

Lastly, the complexity of the mechanisms underlying a hospitalization event should be taken into account. Interestingly, it has been previously written that “Patients are readmitted, and not diagnoses” [46]. Not only biological factors such as inadequate treatment, comorbidities, and progression of disease can deeply affect the rate of hospitalization, but also a number of actors –hospital and primary care physicians, outpatient caregivers, the patients themselves, and specific interventions/organizational characteristics – can exert a strict impact on hospitalization rates. For example, in a large study about HF hospitalizations in the United States, where the total number of HF-related hospitalizations significantly increased from 2001 to 2009, primary HF hospitalizations steadily decreased, whereas the total number of secondary HF hospitalizations increased by nearly 400,000 [47]. The authors argued about the hypothesis that these findings might be related to shifting in coding practices, also because of incentives, as it happened for the downcoding of pneumonia hospitalizations [48]. Besides opportunistic behaviours by hospital coders, it must be recognized that “hospitalizations for HF does not equate to hospitalizations because of HF” [49].

Some limitations should be highlighted in this study. Because the enrolment in the CCM program required that the patient was able to adequately follow the clinical visitations scheduled in the program, the possibility of a selection bias - related to the potential enrolment of patients with lower severity of HF - should be considered. This potential selection bias could have favoured a reduction in mortality in the CCM group. Due to the administrative nature of the data, we were not able to consider some potentially relevant clinical variables in the analysis. Then, we cannot exclude that the observed differences in mortality and hospitalization between the CCM group and the controls were influenced by other covariates. In this regard, information on HF degree measures, co-medications, other disease severity measures, adherence to therapy and discharge plans was not available. It should also be highlighted that, in this study, we did not adjust for the matched pair design, since we only would balance the two groups. Moreover, despite the matching procedure, we decided to use the covariates in multivariable analyses because we were interested in exploring the effect of each single variable. The generalisability of the findings should also be considered with caution. Lastly, our CCM program hinged on general physicians and dedicated nurses. As stated above, a multidisciplinary approach involving other professional figures would be largely preferable for a complex disease such as chronic HF, particularly considering the clinical relevance of comorbidities in the practical management of these subjects. Such a multidisciplinary strategy could also be useful to provide a larger number of diagnostic and therapeutic pathways to the referring general physician, with a potential beneficial impact on the need of hospitalization.

## Conclusions

In conclusion, the implementation of a regional health care program for patients with chronic HF, based on a proactive CCM strategy carried out in a primary care setting, finally yielded a higher risk of HF hospitalization and an improved survival. These findings might have implications for the potential improvement of similar CCM-based programs, highlight the importance of a critical assessment of hospitalization as a measure of outcome, and might support the need for multidisciplinary strategies aimed at optimizing the management of CCM patients in terms of hospitalizations.

## Abbreviations

CCM: Chronic care model
GP: General practitioner
HF: Heart failure
HR: Hazard ratio
IRR: Incidence rate ratio
